# The effect of single biome occupancy on the estimation of biome shifts and the detection of biome conservatism

**DOI:** 10.1371/journal.pone.0248839

**Published:** 2021-03-30

**Authors:** Esther E. Dale, Matthew J. Larcombe, William G. Lee

**Affiliations:** 1 Manaaki Whenua—Landcare Research, Dunedin, New Zealand; 2 Department of Botany, University of Otago, Dunedin, New Zealand; 3 School of Biological Sciences, University of Auckland, Auckland, New Zealand; CNRS - Universite de Pau et des Pays de l’Adour - E2S UPPA, FRANCE

## Abstract

Biome conservatism is often regarded as common in diversifying lineages, based on the detection of low biome shift rates or high phylogenetic signal. However, many studies testing biome conservatism utilise a single-biome-per-species approach, which may influence the detection of biome conservatism. Meta-analyses show that biome shift rates are significantly lower (less than a tenth), when single biome occupancy approaches are adopted. Using New Zealand plant lineages, estimated biome shifts were also significantly lower (14–67% fewer biome shifts) when analysed under the assumption of a single biome per species. Although a single biome approach consistently resulted in lower biome shifts, it detected fewer instances of biome conservatism. A third of clades (3 out of 9) changed status in biome conservatism tests between single and multiple biome occupancy approaches, with more instances of significant biome conservatism when using a multiple biome occupancy approach. A single biome approach may change the likelihood of finding biome conservatism because it assumes biome specialisation within species, falsely recognises some biome shift types and fails to include other biome shift types. Our results indicate that the degree of biome fidelity assumed has a strong influence on analyses assessing biome shift rates, and biome conservatism testing. We advocate analyses that allow species to occupy multiple biomes.

## Introduction

Biome conservatism, also known as phylogenetic biome conservatism [1] or phylogenetic niche conservatism [2], is the tendency of species or lineages to remain in their ancestral biome as they diversify. Biome shifts represent evolutionary changes that allow lineages to overcome biome boundaries [3] and are expected to be rare in lineages exhibiting biome conservatism [1]. Biome conservatism is typically tested through examining phylogenetic signal or estimating biome shift rates. Phylogenetic signal in biomes occupied is quantified to test for phylogenetic clustering, which indicates biome conservatism [4, 5]. The biome shift estimation approach involves comparing observed biome shift rates to a threshold, typically determined by a null simulation, values below which indicates biome conservatism [1, 5–7]. This method depends on assigning all species to a particular biome, frequently a single preferred biome (Table 1), known as the modal biome. Using the modal biome of each species to quantify biome shifts is conceptually simple, but overlooks the range of biomes occupied by many species in natural habitats, albeit with different abundances. Here we consider biome occupancy based on the distribution across the entire niche of the species, encompassing the range of conditions where individuals can establish, grow to maturity and regenerate.

**Table 1:** Clade size, proportion of biome specialists, whether multiple biome occupancy is incorporated into analyses, and biome shift rates for a range of biome shift studies [1, 2, 4–13].

			Clade size^a^	Specialists^b^		Multiple biomes^c^	Biome shifts		
Study	Location	Lineage		No.	%		Count^d^	Rate^e^	Description^f^
Cardillo et al. 2017	Australia	*Hakea*	151	113	75	Yes	47	0.31	frequent
Crisp et al. 2009	Southern Hemisphere	45 taxa	~11,000	-	-	No	396	0.04	rare
Cruz et al. 2017	Brazil	*Cryptanthus*	48	-	-	No	4	0.08	rare
Gagnon et al. 2019	Global	Caesalpinia Group	168	144	86	Yes	24	0.14	strong conservatism
Gamisch et al. 2016	Madagascar	*Bulbophyllum*	30	26	87	Yes	21	0.7	exceptionally high
Holstein & Renner 2011	Africa	*Coccinia*	27	-	-	No	6	0.22	frequent
Jara-Arancio et al. 2014	Western South America	*Leucornye*	17	13	76	No	2	0.11	low conservatism
Simon et al. 2009	South America	*Andira*	25	20	80	No	2	0.04	frequent
Simon et al. 2009	South America	*Lupinus*	94	93	99	No	1	0.01	frequent
Simon et al. 2009	South America	*Mimosa*	255	223	87	No	11	0.04	frequent
Simon et al. 2009	South America	Microlicieae	60	54	90	No	1	0.02	frequent
Spriggs et al. 2015	Northern Hemisphere	*Viburnum*	138	-	-	No	10+	0.07	more common [than expected]
Toon et al. 2015	Australia	Triondiinae	66	51	77	Yes	15+	0.23	multiple
Weeks et al. 2014	Global	Anacardiaceae	169	164	97	Yes	74	0.44	extremely common
Weeks et al. 2014	Global	Burseraceae	136	132	97	Yes	11	0.08	few
Zizka et al. 2020	The Tropics	Bombacoideae	174	73	42	Yes	84	0.48	common
This study	New Zealand	*Chionochloa*	22	12	55	Yes	23	1.04	
This study^g^	New Zealand	*Coprosma*	54	30	56	Yes	40	0.74	
This study	New Zealand	*Melicytus*	11	7	64	Yes	6	0.54	
This study	New Zealand	*Myrsine*	11	5	45	Yes	6	0.54	
This study	New Zealand	*Poa* X	29	20	69	Yes	21	0.72	
This study	New Zealand	*Pseudopanax*	13	4	31	Yes	7	0.54	
This study	New Zealand	*Rytidosperma* A	7	3	43	Yes	9	1.29	
This study	New Zealand	*Rytidosperma* B	7	4	57	Yes	6	0.86	
This study^h^	New Zealand	*Veronica*	51	23	45	Yes	40	0.78	

^a^ Clade size is number of taxa

^b^ Specialists is the number and percentage of species that occupy a single biome

^c^ Multiple biomes is whether analyses incorporate multiple biome occupancy

^d^ Biome shift counts were rounded down to the nearest whole number

^e^ Biome shift rate is the number of biome shifts divided by the number of species in a clade

^f^ Description is an in-text description of biome shift rate directly quoted from the original studies.

^g^ used phylogeny from [14]

^h^ used phylogeny from [15].

The single biome occupancy approach is common in part because it was adopted by early studies which indicated that biome conservatism is widespread [1]. Methodologically, some analyses require a single biome per species (e.g. some phylogenetic signal [4] or ancestral state reconstruction methods [8]) which constrains options. This is satisfactory in lineages where all species are biome specialists, meaning they each occupy a single biome, but for many other lineages where species naturally occur in multiple biomes, a single biome per species approach can be problematic. The issue is that assumptions of single biome occupancy, or exclusion of species that do not conform to this assumption, may introduce bias towards detection of biome conservatism.

There are many lineages with species that occur in multiple biomes with typical values of between 10–25% of species [4–6, 9, 10], that can range as high as 91% [16]. Biome shift estimates inferred with a single biome occupancy approach may not detect some types of biome shift, for example when species expand into another biome while retaining occupancy in the ancestral biome. Before biome conservatism becomes the dominant paradigm in evolutionary ecology, the influence of the assumption of single biome occupancy needs to be evaluated.

We hypothesise that a single biome occupancy approach results in fewer estimated biome shifts than a more realistic multiple biome approach, causing a bias towards the detection of biome conservatism. We expect that the effect of the single biome occupancy approach will be less pronounced in lineages with a high proportion of species that occur in a single biome (biome specialists), because fewer species would have a change in their biome occupancy when data is converted to a single biome per species for analyses. In contrast, the single biome occupancy approach may have a larger effect on lineages where almost all species occur in multiple biomes, because most species would have the biomes that they occupy modified to a single biome for the analysis.

We test these hypotheses by examining published studies and analysing nine New Zealand plant lineages estimating biome shifts and quantifying phylogenetic signal using both single biome and multiple biome approaches.

## Materials and methods

Data from 25 clades in previously published biome shift studies were compiled including the number of species per lineage (clade size), number of biome shifts (biome shift count), number of species that occur in a single biome (specialists), and whether analyses accommodated multiple biome occupancy of species (multiple biomes). Biome shift rates were calculated as the number of biome shifts divided by the number of species in the lineage. The 25 lineages from biome shift studies were initially compiled by examining the references cited in a review paper [3] and any other papers on biome shifts we could find using keyword searches including “biome*”, “biome shift*”, “conservatism”, “transition”, “lineage”, and “phylogenetic niche conservatism”. Our initial list was 41 papers, 11 of which were excluded as not being relevant based on their abstracts, a further 17 papers were excluded after reading the full text. Papers were excluded if they did not estimate biome shifts using a quantitative method, did not include the total number of species in the lineage, and omitted to describe whether a single or multiple biome occupancy approach was used in the biome estimation analyses. Thirteen papers with a total of 25 lineages were selected for our analyses based on these criteria. We did not exclusively use a database searching approach to find these studies because we wanted to include early work in this still-developing field, before the concept of “biome shifts” was formalised.

We selected New Zealand as the study system for a more comprehensive analysis because of the availability of biome occupancy data and phylogenies for nine lineages [14, 15, 17], and the biogeographic context. The available data enabled us to run analyses using both single and multiple biome occupancy approaches then compare findings. Uncertainties around the historical distribution of biomes can pose a problem for estimating lineage-level biome shift rates [18]. For example, in many areas dating the emergence and evolution of different biomes remains difficult [19], which makes modelling historic biome occupancy challenging, resulting in compounding uncertainties in biome shift estimates. New Zealand has long been of interest to biogeographers because of its isolation from other Gondwanan landmasses for the last 80 million years [20]. With an independent evolutionary history, supplemented by long distance immigration of biota, especially since the Miocene [21], and a relatively recent human history (since 13th Century [22]), New Zealand maintains many natural spatial patterns reflecting both biogeographic history and ecological sorting processes. Although forest biomes have pre-dominated under warm and cool temperate climates for most of New Zealand’s history, they have been periodically limited in the last 5 million years as mountains formed and glaciations occurred [23]. These more recent non-forest biomes, both above and below tree line, have been critical zones for the diversification of the New Zealand flora and can be reliably dated to coincide with the uplift of the Southern Alps [24]. The New Zealand context is an ideal study system for comparing biome shift methods because the relatively isolated system, well-sampled lineages, few biomes, clear sequence of biome availability, and reliable biome emergence dates, which minimise the context-associated uncertainties that accompany biome shift estimation.

The biome concept, emphasising physiognomically similar vegetation types [25], can be readily applied to New Zealand, distinguishing Forest, Open or non-forest habitats below treeline, and the Alpine. New Zealand presents an excellent opportunity for examining biome shifts, and therefore biome shift analysis methods, because the appearance of novel biomes is closely linked to uplift of the Southern Alps, which has resulted in a sequence of different biomes appearing sequentially through time. The Forest biome is the oldest New Zealand biome, and was present before New Zealand separated from Gondwana [26]. The Open biome has been a feature since 4 Ma ago, and Alpine has been present for 1.9 Ma and has covered large areas most recently in the last 0.9 Ma [24]. These biomes encompass all New Zealand vegetation types and habitats [20]. Open habitats currently include both primary and secondary grassland and shrubland and are associated with climate, soil, and disturbance conditions that limit forest establishment.

The nine focal New Zealand clades cover a range of colonisation dates with crown ages, a proxy for colonisation date [27], ranging from 2–40 Ma (Table 2) covering the availability of different biomes. *Chionochloa*, *Coprosma*, *Melicytus*, *Myrsine*, *Pseudopanax*, *and Veronica* colonised when forests were widespread while *Poa* X, and both *Rytidosperma* clades arrived in New Zealand after the open biome emerged but before the alpine appeared [24].

**Table 2:** Focal New Zealand clades by number of taxa, stem age, crown age, diversification rate and life form.

	Number of taxa					
Clade	Total	Included	Stem age	Crown age	Diversification rate	Life form
*Chionochloa*	23	22	19.95 (23.60–16.65)	8.60 (13.95–4.50)	0.16 (0.13–0.19)	Grass
*Coprosma*	55	54	25.48 (30.50–23.10)	13.73 (18.40–9.10)	0.16 (0.13–0.17)	Woody
*Melicytus*	14	11	28.21 (33.6–23.2)	11.02 (11.6–7.4)	0.09 (0.08–0.11)	Woody
*Myrsine*	11	11	11.81 (12.3–3.5)	9.98 (10.8–3.1)	0.2 (0.19–0.69)	Woody
*Poa* X	29	29	3.55 (5.50–1.80)	Not reported	0.95 (0.61–1.87)	Grass
*Pseudopanax*	13	13	39.8 (45.5–20.8)	39.78 (44.1–22.8)	0.06 (0.06–0.12)	Woody
*Rytidosperma* A	7	7	3.85 (5.55–2.35)	3.10 (4.55–1.75)	0.5 (0.35–0.83)	Grass
*Rytidosperma* B	7	7	Not reported	2.00 (3.45–0.85)	0.63 (0.36–1.47)	Grass
*Veronica*	124	51	10.21 (13.33–7.21)	7.62 (10.26–5.29)	0.47 (0.36–0.67)	Woody

Number of taxa—Total equals the number of taxa in the clade indigenous to New Zealand. Number of taxa—Included is the number of taxa included in biome shift analyses. Stem and crown ages are in Ma with ± SD or 95% highest posterior density interval in parentheses, dates were sourced from [28] (*Chionochloa*, *Coprosma*, *Poa* X, *Rytidosperma* A, *Rytidosperma* B, and *Veronica*) or [17] (*Melicytus*, *Myrsine*, and *Pseudopanax*). Diversification rates are species per Ma, calculated using the Magallón and Sanderson [29] method for stem ages, except for the *Rytidosperma* B diversification rate which was based on crown age because there was no stem age available.

Occupancy of each species in Forest, Open and Alpine biomes was determined based on the descriptions of their distributions in the literature [30–42]. A species had to consistently occur in and reach maturity in a biome for it to be counted as occupying that biome, vagrant or seedling occurrences were excluded. We defined Forest as any closed canopy vegetation made up of trees, including regeneration gaps within forest. Open was any vegetation below treeline without a closed tree canopy, and included scrubland, herbfield, and grassland. Alpine was any vegetation above treeline. For each of the taxa in these clades we identified the modal biome as the biome that makes up the largest proportion of its range.

Biome shift rates were estimated by fitting six different biogeographic models to each of our focal clades using the *BioGeoBEARS* R package [43]: DEC, DEC+J, DIVALIKE, DIVALIKE+J, BAYAREALIKE and BAYAREALIKE+J. Each model was fitted using biomes as “areas” and was time-stratified based on the dates of Heenan and McGlone [24]: Forest was always available, Open from 4 Ma and Alpine only since 1.9 Ma. The input files used for this analysis are publicly available [44]. We identified the best model for each clade using a one-tailed chi-squared test to compare models to their +J counterparts (e.g. DEC to DEC+J), and then used AIC to determine which of the model types fitted best. We estimated possible biome occupancy histories for each clade by conducting 100 runs of Biogeographic Stochastic Mapping with *BioGEOBEARS* using both the multiple biome occupancy and single biome per species approaches.

### Hypothesis testing

We compared biome shift rates of studies derived from either a single biome occupancy approach or a multiple biome occupancy approach, for 25 published lineages, including the nine from New Zealand. To make the biome shift rates of the New Zealand clades comparable to the other published studies we counted multiple biome shifts on the same branch as a single biome shift.

Biome shifts between single and multiple biome approaches for all clades were compared using a one-tailed t-test to test whether there were significantly fewer biome shifts estimated with a single biome occupancy approach. Results were qualitatively the same with and without using the New Zealand clades (S1 Table).

The relationship between the percentage of biome specialists (species which occur in a single biome) and the biome shift rate of clades was tested using a simple linear regression model to examine whether estimated biome shift rates were lower in lineages with a higher proportion of biome specialists. Only published studies which reported the proportion of biome specialists, or data that made it possible to infer the proportion of biome specialists, were included in this analysis; nine lineages were from studies that used a single biome occupancy approach, and sixteen from studies that used a multiple biome occupancy approach. Model assumptions were tested by examining the residuals and a normal-quantile-quantile plot.

We compared the biome shift rates between the single and multiple biome occupancy approaches using the nine New Zealand clades in which biome shifts rates had been estimated with both approaches using a one-tailed paired t-test. We quantified the proportional decline in biome shift rate to determine the magnitude of the change in estimated biome shifts between the single and multiple biome approaches in a way that was comparable between lineages. A high proportional decline in biome shift rate would indicate a large drop in estimated biome shifts when a single biome occupancy approach is used, and a low proportional decline indicates little effect of the single biome occupancy assumption. We calculated the proportional decline in biome shift rate as follows: *B*_*decline*_ as the proportional decline in biome shifts, *B*_*multiple*_ as the biome shift rate estimated using a multiple biome occupancy approach, and *B*_*single*_ as the equivalent using a single biome occupancy approach: $B_{decline}=\frac{B_{multiple}-B_{single}}{B_{multiple}}$.

We tested the significance of the relationship between the proportional decline in biome shift rate with the percentage of biome specialists in a clade using simple linear regression to test whether there was a lower proportional decline in biome shift rate for lineages with more biome specialists. Model assumptions were tested by examining residuals vs fitted values and normal-quantile-quantile plots.

Biome conservatism was identified in the New Zealand clades using biome shift rates and phylogenetic signal to examine whether biome conservatism was more frequently detected when a single biome occupancy approach was used compared to a multiple biome occupancy approach. For biome shift rates we used the method of Crisp et al. [1] which involved estimating biome shift rates using the observed biome occupancy data and comparing it to biome shift rates of 1000 simulations with randomised biome occupancy states. For each randomised phylogeny we completed a Biogeographic Stochastic Mapping run using the same model that fitted the observed data best and then quantified biome shift rates. Biome conservatism was indicated if > 0.95 of the 1000 runs had greater biome shift rates than the observed biome shift rate for that clade. All these biome conservatism tests used biome shift counts simplified in the same manner as the meta-analysis with 25 clades described above. Repeating this biome conservatism test using raw biome shift estimates demonstrated that this simplification did not significantly influence the outcome (S2 Table).

Phylogenetic signal in biomes occupied is typically analysed by calculating Pagel’s λ [e.g. 4, 5], however it requires a single discrete character state per species, so is not possible to use with multiple biome occupancy. Often species that occur in multiple biomes are excluded [45] or only the modal biome is used [4]. We tested phylogenetic signal in the occupancy of each biome separately (Forest, Open, Alpine) because this can accommodate both single and multiple biome occupancy approaches. We used the *D* statistic, a phylogenetic signal metric for binary traits, calculated and significance-tested using the ‘phylo.d’ function in the *caper* package [46]. The *D* statistic is equal to 1 if the distribution of the trait at the tips is phylogenetically random, low or negative *D* values indicate phylogenetic conservatism, and values greater than 1 show over-dispersion [47]. A *D* value significantly lower (p<0.05) than expected under a random situation (where *D* = 1) would indicate phylogenetic clustering in occupancy of that biome, and therefore biome conservatism. We used this phylogenetic signal approach to test for biome conservatism in both single and multiple biome occupancy data.

## Results

The best-fitting BioGeoBEARS models for the single biome occupancy approach were DEC (*Rytidosperma* A and B), DEC+J (*Chionochloa*, *Coprosma*, *Melicytus*, *Poa* X, *Pseudopanax*, and *Veronica*), and DIVALIKE (*Myrsine*). For the multi ple biome occupancy approach, the best-fitting models were DEC (*Melicytus*, *Myrsine*, and *Rytidosperma* A), DEC+J (*Chionochloa*, *Poa* X, and *Rytidosperma* B), BAYAREALIKE (*Pseudopanax*), and BAYAREALIKE+J (*Coprosma* and *Veronica*).

**Fig 1:**
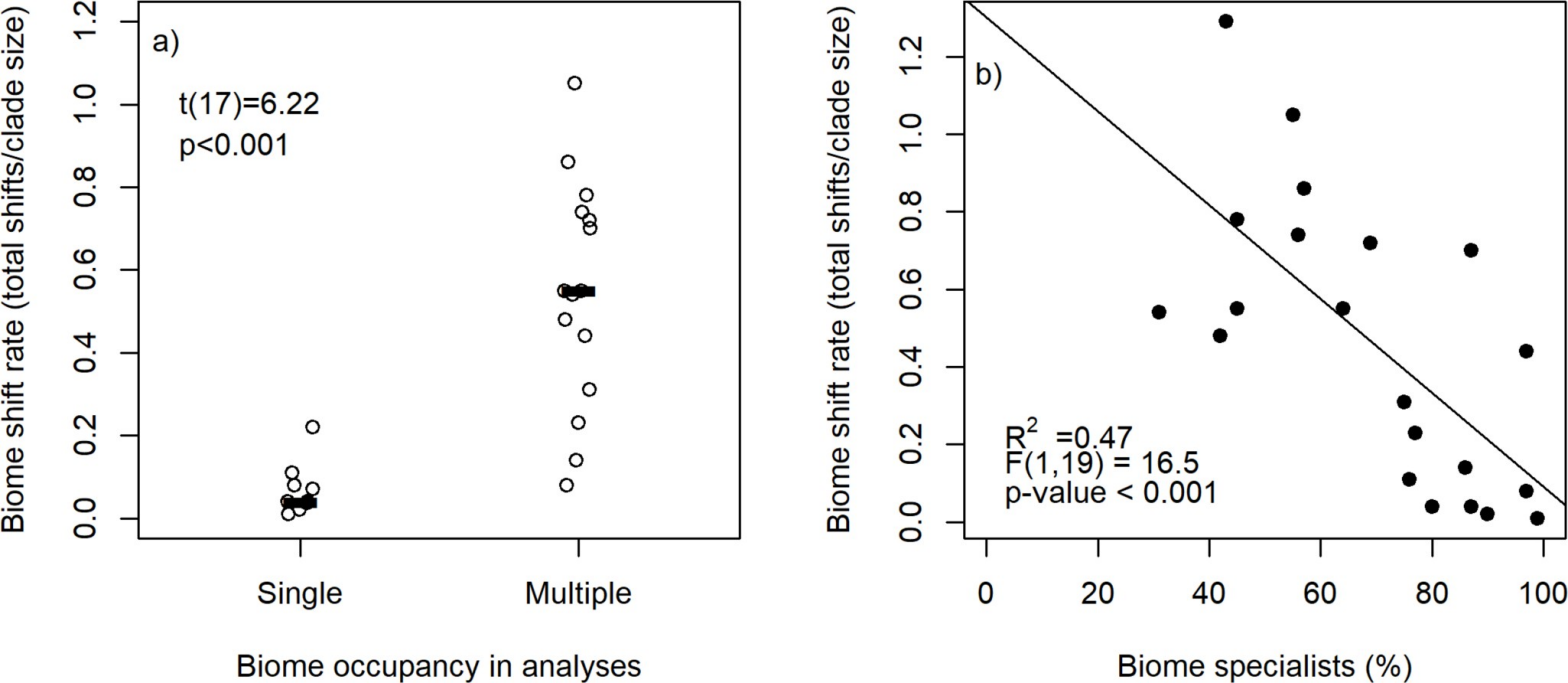
Biome shift rates for clades a) analysed with single or multiple biome occupancy per species approach, and b) by the percentage of species that occur in a single biome. Each point represents a clade in a published study, see Table 1 for information on each clade. Black rectangles indicate the median biome shift rate across all studies. The t-test results are for a one-tailed test comparing biome shift rates in studies that utilised a single biome occupancy approach to studies with analyses that accommodate occurrence in multiple biomes. Model outputs on b) are for a simple linear regression model.

Biome shift rates were significantly lower with single compared to a multiple biome occupancy approach in all 25 published lineages, including the nine New Zealand lineages (Fig 1A). The median shift rate of single biome occupancy studies (0.04, quartiles 0.08–0.36) was less than a tenth of equivalent rates in multiple biome occupancy studies (0.55, quartiles 0.4–0.75). These results remained significant when we restricted comparisons to the non-New Zealand clades (one-tailed t-test t(7) = 3.20, p<0.01). Biome shift rate declined significantly as the percentage of biome specialists in clades increased (Fig 1B).

For the New Zealand clades, where we could directly compare single and multiple biome approaches, biome shift rates were significantly lower when analysed with a single biome approach (Table 3, paired t-test t(8) = 3.208, p = 0.003). The dominance of biome specialists in a lineage does not influence this result because proportional differences in biome shift rates between the two types of occupancy analyses were unrelated to the percentage of biome specialists (R^2^ = 0.05, F(1,7) = 0.33, p = 0.58).

**Table 3:** Mean biome shift rates and associated biome conservatism test results for both the single biome occupancy approach (“Single”) and the multiple biome occupancy approach (“Multiple”) for the New Zealand lineages.

	Mean biome shift rate (± standard deviation)		Proportion of null simulations greater than observed biome shifts		Absolute (percentage) change in tendency towards biome conservatism
Clade	Single	Multiple	Single	Multiple	
*Chionochloa*	0.73 (± 0.05)	1.05 (± 0.07)	0.40	0.44	+0.04 (10%)
*Coprosma*	0.56 (± 0.04)	0.74 (± 0.05)	0.58	0.85	+0.27 (47%)
*Melicytus*	0.18 (± 0.03)	0.55 (± 0.07)	0.98*	0.98*	0.00 (0%)
*Myrsine*	0.27 (± 0.06)	0.55 (± 0.05)	0.00	0.35	+0.35 (N/A)
*Poa* X	0.62 (± 0.05)	0.72 (± 0.07)	0.69	0.95*	+0.26 (38%)
*Pseudopanax*	0.31 (± 0.05)	0.54 (± 0.04)	0.83	0.32	-0.51 (61%)
*Rytidosperma* A	0.86 (± 0.13)	1.29 (± 0.16)	0.81	0.22	-0.59 (73%)
*Rytidosperma* B	0.57 (± 0.12)	0.86 (± 0.14)	0.58	0.89	+0.31 (53%)
*Veronica*	0.49 (± 0.04)	0.78 (± 0.05)	0.70	0.56	-0.14 (20%)

Null simulations were conducted for each clade by randomising biomes occupied, with 1000 replicates per clade. Biome conservatism is considered significant (*) if 0.95 or greater of the simulations have more biome shifts than the observed biome shift count. The change in tendency towards biome conservatism comparing single with multiple biome occupancy approaches is indicated, positive values demonstrate an increase in tendency towards biome conservatism, negative values demonstrate a decrease in tendency towards biome conservatism.

More clades exhibited significant biome conservatism with the multiple biome occupancy approach than the single biome occupancy approach for both methods. With the biome shift rates method, four clades exhibited a noticeably greater (>25%) shift in tendency towards biome conservatism using the multiple biome approach and rates-based method (*Coprosma*, *Myrsine*, *Poa* X, *Rytidosperma* B, Table 3) but this change was only significant for *Poa* X (Table 3). *Melicytus* exhibited no change while two clades (*Pseudopanax*, *Rytidosperma* A) showed a notable (>25%) decline in tendency towards biome conservatism between single and multiple biome approaches (Table 3). With the phylogenetic signal method, more clades and biomes exhibited significant biome conservatism with the multiple biome occupancy approach than the single biome occupancy approach (Table 4), refuting our hypothesis of greater bias towards biome conservatism when a single biome occupancy approach is used. *Melicytus* had significant *D*-values for Forest and Open biomes for both biome occupancy approaches (Table 4), consistent with the biome conservatism test results based on biome shift rates (Table 3). *Coprosma* exhibited a significant *D*-value for just Alpine when using the single biome approach, but all three biomes with the multiple biome occupancy approach (Table 4). This contrasts with no evidence for biome conservatism based on the biome shift rates biome conservatism test, but does fit with the 47% increase in tendency towards biome conservatism (Table 3). *Veronica* had significant a *D*-value for only Alpine under the single biome approach, but both Forest and Alpine under the multiple biome occupancy approach, these contrast with the lack of biome conservatism and decline in tendency towards biome conservatism detected in *Veronica* using the biome shift rates method. The *D*-values were not significant for any biomes or either biome occupancy method for *Chionochloa*, *Myrsine*, *Poa* X, *Pseudopanax*, *Rytidosperma* A, and *Rytidosperma* B (Table 4).

**Table 4:** Phylogenetic signal in biome occupancy of the New Zealand lineages for single biome occupancy (“Single”) and multiple biome occupancy (“Multiple”) approaches.

		*D* value	
Clade	Biome	Single	Multiple
*Chionochloa*	Forest	-0.28	1.28
*Chionochloa*	Open	1.18	1.22
*Chionochloa*	Alpine	1.15	1.50
*Coprosma*	Forest	0.62	0.55*
*Coprosma*	Open	0.69	0.29*
*Coprosma*	Alpine	-0.08*	0.22*
*Melicytus*	Forest	-2.58*	-1.75*
*Melicytus*	Open	-2.77*	-2.47*
*Melicytus*	Alpine	NA	2.45
*Myrsine*	Forest	0.82	-1.45
*Myrsine*	Open	1.24	1.43
*Myrsine*	Alpine	-1.19	-1.00
*Poa* X	Forest	-4.83	0.77
*Poa* X	Open	1.02	1.12
*Poa* X	Alpine	1.01	0.72
*Pseudopanax*	Forest	0.67	NA
*Pseudopanax*	Open	0.65	1.14
*Pseudopanax*	Alpine	NA	1.36
*Rytidosperma* A	Forest	NA	3.20
*Rytidosperma* A	Open	0.17	-0.17
*Rytidosperma* A	Alpine	0.12	0.30
*Rytidosperma* B	Forest	NA	NA
*Rytidosperma* B	Open	2.94	2.58
*Rytidosperma* B	Alpine	2.6	0.61
*Veronica*	Forest	1.07	0.40*
*Veronica*	Open	0.65	1.21
*Veronica*	Alpine	0.39*	0.48*

*D* values that were significantly different to the random simulation (p<0.05) are indicated with an *, they indicate biome conservatism.

## Discussion

A single biome occupancy approach was associated with fewer biome shifts than a multiple biome occupancy approach, both in the broader meta-analysis of published studies (Fig 1A) and the more detailed analysis of the New Zealand clades. This supports our hypothesis of markedly fewer estimated biome shifts with a single compared to multiple biome occupancy approach. Lineages with a higher percentage of biome specialists tended to exhibit lower biome shift rates (Fig 1B), however we observed that the magnitude of the effect of this assumption on biome shift estimates was not related to the degree of biome specialisation in the lineage. This indicates that the assumption of single biome occupancy is equally problematic for determining biome shift rates in lineages regardless of the proportion of biome specialists. Even in lineages with a high degree of biome specialisation, species in multiple biomes may reflect an intermediate stage of range expansion between two biomes [4], or the beginnings of ecological speciation [9]. Therefore, to exclude species occupying multiple biomes or treat them as if they occupy a single biome, even if there are relatively few of them in a lineage, could be potentially misleading.

Statistically significant biome conservatism was detected marginally more frequently when a multiple biome occupancy approach was used for both biome conservatism testing methods (Tables 3 and 4). This disagrees with our hypothesis of a single biome approach causing bias towards detection of biome conservatism. However, the effect of the single biome assumption on the direction of change in tendency towards biome conservatism was variable. Five lineages had an increase in tendency towards biome conservatism with a multiple biome occupancy approach (*Chionochloa*, *Coprosma*, *Myrsine*, *Poa* X, and *Rytidosperma* B), three had a decrease in tendency towards biome conservatism (*Pseudopanax*, *Rytidosperma* A, and *Veronica*), and one exhibited no change (*Melicytus*).

**Fig 2:**
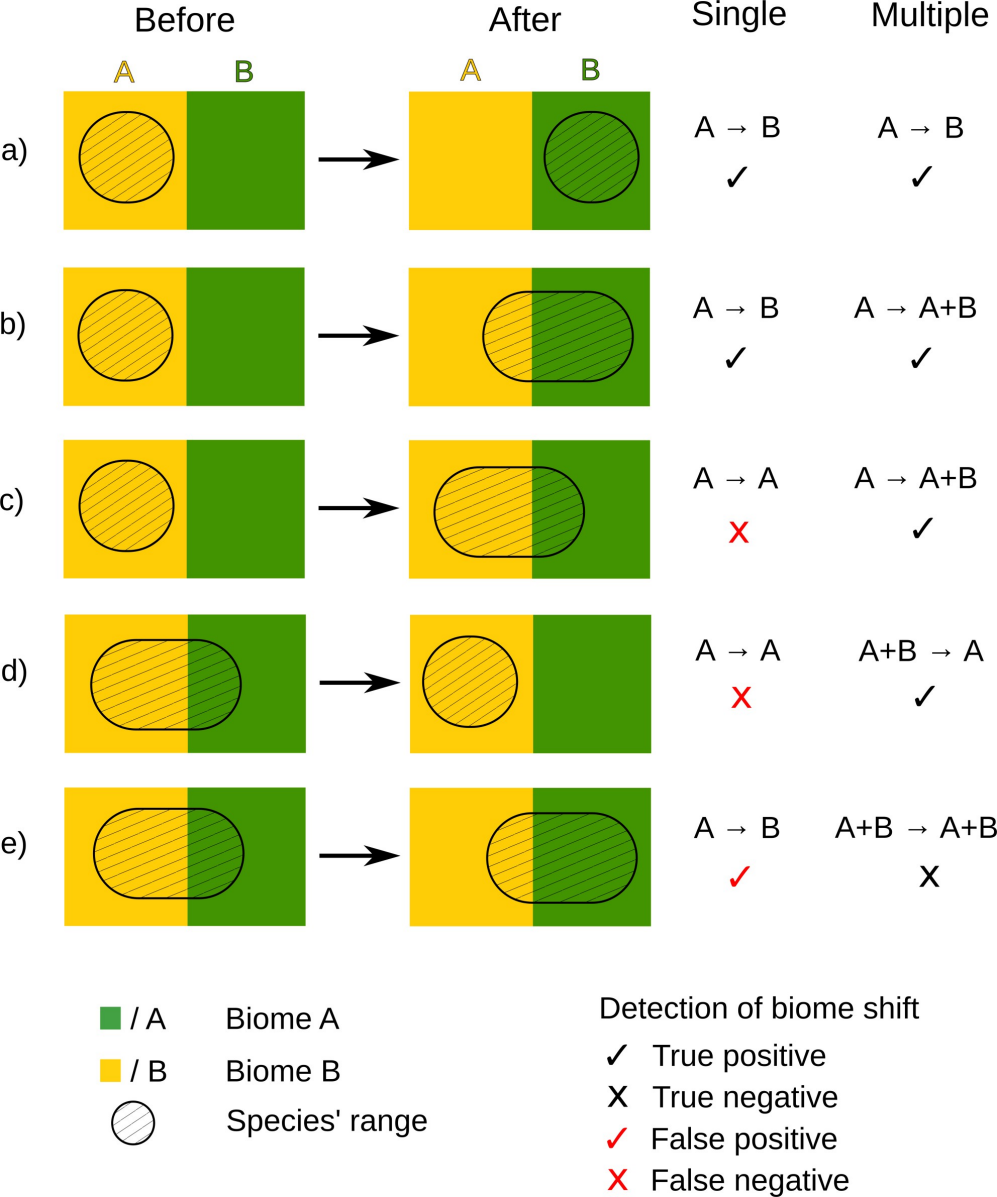
Types of biome shift and how they would be recognised under a single or multiple biome occupancy per species approach. A (yellow) and B (green) are two different biomes, black cross-hatched areas indicate the distribution of a species, arrows indicate passing of time, ticks show a detected biome shift, and crosses show a lack of a detected biome shift. True positives or negatives are shown in black, and false positives or negatives are in red.

Variation in the effect of the single compared to multiple biome occupancy approach on the detection of biome conservatism is likely a result of single biome occupancy failing to recognise some types of biome shifts and falsely including other types of biome shifts (Fig 2). True positive biome shifts are only detected under a single biome approach if the biome shift also involves a change in the modal biome—the biome most frequently occupied across the range of the species. This is satisfactory for biome switches (Fig 2A) and range expansion shifts where the modal biome also changes (Fig 2B). However, biome shifts are not detected under the single biome occupancy approach when there is a range expansion into a new biome that only forms a small part of the species’ new range, resulting in a false negative (Fig 2C). Similarly, when there is a range reduction out of a biome but no change in modal biome then there is also a false negative biome shift (Fig 2D). We consider these false negatives because they represent true biome shifts, in which lineages either overcome biome boundaries or become more specialised [3], but are not able to be detected under a single biome occupancy approach. False positives also occur with the single biome approach when an apparent biome shift is detected for a species that already occurs in multiple biomes but there is a change in its modal biome (Fig 2E). The greater number of clades that exhibited a change to biome conservatism or a notable increase (>25%) in tendency towards biome conservatism between single and multiple biome occupancy approaches (4 clades), suggest that false negatives are more likely to be an issue than false positives. These false negatives and false positives may also be an issue in studies that allow for multiple biome occupancy but have a relatively high occurrence threshold for determining presence in a biome (e.g. a third of the area of the niche in a biome [9]). This is essentially the same assumption as a single biome approach: that occupancy in a biome is only valid if it makes up a high proportion of a species’ distribution.

There are merits and limitations to both methods of testing biome conservatism. The difference in biome conservatism results between the biome shifts rates method and the phylogenetic signal method likely reflects the difference in grouping of biomes. The biome shift rates approach considers all biomes and shifts between them collectively, while the phylogenetic signal method examines conservatism in occurrence each biome separately. This independent treatment of different biomes with the phylogenetic signal approach may be useful in being able to isolate different degrees of conservatism for different biomes. *Veronica* for example, exhibited significant phylogenetic signal in occupancy of Forest and Alpine, indicating within-biome diversification in both these two biomes, but not the Open biome. Perhaps the lack of within-biome diversification in the Open biome has resulted in sufficiently high biome shift rates in *Veronica* to yield a non-significant biome conservatism result using the biome shift rate method. However, considering biomes individually comes at the cost of an integrated assessment of biome conservatism. Phylogenetic signal of occupancy in each biome is also limited by its inability to work in cases where all species in a lineage occur or are absent for a particular biome. For example, *Pseudopanax* has every species occurring in Forest, to some extent, and therefore exhibits complete conservatism in occupancy of Forest, its ancestral biome, but it is not detected as such. Both the phylogenetic signal method and the biome shift rate method for testing biome conservatism have merits and highlight different aspects of biome conservatism, suggesting it would be best to use both methods for a more complete understanding.

There is no consensus in the literature on what biome shift rates are considered rare or frequent, which makes rate-based determination of biome conservatism difficult. For example, biome shift rates of 0.04 have been considered both “rare” [1] and “frequent” [2], while others suggested that a biome shift rate of 0.22 was “frequent”, despite being fewer than was expected by chance [7]. Other studies do not provide qualitative assessments, referring to a rate of 0.23 as simply “multiple” biome shifts [10] (Table 1). This lack of consensus may in part reflect the changing view of the relative frequency of biome shifts as this field has developed. Any biome shifts might have been considered significant when the consensus was that they occurred rarely, but now that more examples are emerging of clades with high biome shift rates and low biome specialisation [16], a more conservative language may be evolving. It may also reflect the context-dependence of assessing biome shift rates, because there are many factors which influence rates including lineage size, number of biomes present, length of biome boundaries, biome age, environmental similarity of biomes, and methodological approaches [1, 3]. It is therefore important to have a quantitative method for testing biome conservatism, rather than using absolute biome shifts rates.

We had predicted that lineages with a higher proportion of biome specialists would be less impacted by a single biome per species analysis approach, but we did not find support for this. However, the effect of assuming single biome occupancy may differ depending on the number of biomes in a system and diversification patterns. More biomes may be present due to inherent habitat diversity, or by using a finer scale biome typology, both of which are likely to exhibit more biome shifts than in a system with fewer biomes [5]. Studies in systems with many biomes are likely to be more biased by the assumption of single biome occupancy than systems with few biomes, because multiple biome occupancy and biome shift rates both increase as the number of biome boundaries increases [3].

## Conclusions

Our predication that single biome occupancy assignment would result in bias towards detecting fewer biome shifts was supported. This pattern of lower biome shift estimates when using a single biome per species approach persisted even in lineages with a high proportion of species specialised to a single biome. However, the effect of using a single biome per species approach on detecting biome conservatism was the opposite to our expectation of bias towards conservatism when a single biome approach was used. A third of clades (3/9) had some change in biome conservatism results between the single and multiple biome occupancy approaches, with more biome conservatism detected when the multiple biome occupancy approach was used. We suggest that this change in responses of lineages’ tendency towards biome conservatism, when using a single compared to a multiple biome per species approach, may be due to the failure to detect some types of biome shift and incorrect inclusion of apparent biome shifts.

We advocate that the multiple biomes occupancy should be included in analyses of biome conservatism.

## Supporting information

S1 Checklist PRISMA 2009 checklist. (DOC)

S1 Table T-test results when the New Zealand clades are excluded. A one-tailed t-test testing the significance of the difference in biome shifts between single and multiple biome approaches for all clades, for all clades and excluding the New Zealand clades, to test whether these clades were overly-influencing results. Significant p-values are indicated with an *.
(DOCX)

S2 Table Biome conservatism test results for raw biome shift counts. Phylogenetic null model analysis testing the effect of single versus multiple biome occupancy on biome conservatism with raw biome shift estimates used for both. Null simulations were conducted for each clade by randomising biomes occupied, with 1000 replicates per clade. Biome conservatism is considered significant (*) if 0.95 or greater of the simulations have more biome shifts than the observed biome shift count. A positive/negative change in tendency towards biome conservatism comparing single with multiple biome occupancy approaches is indicated.
(DOCX)

S1 Flow diagram PRISMA 2009 flow diagram. (DOC)

## References

[1] Crisp MD, Arroyo MTK, Cook LG, Gandolfo MA, Jordan GJ, McGlone MS, et al. Phylogenetic biome conservatism on a global scale. Nature. 2009;458(7239):754–6. http://www.nature.com/nature/journal/v458/n7239/suppinfo/nature07764_S1.html. 10.1038/nature07764 19219025

[2] Simon MF, Grether R, de Queiroz LP, Skema C, Pennington RT, Hughes CE. Recent assembly of the Cerrado, a neotropical plant diversity hotspot, by *in situ* evolution of adaptations to fire. Proceedings of the National Academy of Sciences. 2009;106(48):20359–64. 10.1073/pnas.0903410106 19918050 PMC2787167

[3] Donoghue MJ, Edwards EJ. Biome shifts and niche evolution in plants. Annual Review of Ecology, Evolution, and Systematics. 2014;45:547–72.

[4] Cardillo M, Weston PH, Reynolds ZK, Olde PM, Mast AR, Lemmon E, et al. The phylogeny and biogeography of *Hakea* (Proteaceae) reveals the role of biome shifts in a continental plant radiation. Evolution. 2017;71:1928–43. 10.1111/evo.13276 28548206

[5] Gagnon E, Ringelberg JJ, Bruneau A, Lewis GP, Hughes CE. Global Succulent Biome phylogenetic conservatism across the pantropical Caesalpinia Group (Leguminosae). New Phytologist. 2019;222(4):1994–2008. 10.1111/nph.15633 30536385

[6] Jara‐Arancio P, Arroyo MT, Guerrero PC, Hinojosa LF, Arancio G, Méndez MA. Phylogenetic perspectives on biome shifts in *Leucocoryne* (Alliaceae) in relation to climatic niche evolution in western South America. Journal of Biogeography. 2014;41(2):328–38.

[7] Holstein N, Renner SS. A dated phylogeny and collection records reveal repeated biome shifts in the African genus *Coccinia* (Cucurbitaceae). BMC Evolutionary Biology. 2011;11(1):28. 21269492 10.1186/1471-2148-11-28 PMC3041684

[8] Cruz GA, Zizka G, Silvestro D, Leme EM, Schulte K, Benko-Iseppon AM. Molecular phylogeny, character evolution and historical biogeography of *Cryptanthus* Otto & A. Dietr.(Bromeliaceae). Molecular Phylogenetics and Evolution. 2017;107:152–65. 10.1016/j.ympev.2016.10.019 27989631

[9] Gamisch A, Fischer GA, Comes HP. Frequent but asymmetric niche shifts in *Bulbophyllum* orchids support environmental and climatic instability in Madagascar over Quaternary time scales. BMC Evolutionary Biology. 2016;16(1):14. 10.1186/s12862-016-0586-3 26781289 PMC4717530

[10] Toon A, Crisp M, Gamage H, Mant J, Morris D, Schmidt S, et al. Key innovation or adaptive change? A test of leaf traits using Triodiinae in Australia. Scientific Reports. 2015;5:12398. 10.1038/srep12398 26215163 PMC4648476

[11] Spriggs EL, Clement WL, Sweeney PW, Madriñán S, Edwards EJ, Donoghue MJ. Temperate radiations and dying embers of a tropical past: the diversification of *Viburnum*. New Phytologist. 2015;207(2):340–54. 10.1111/nph.13305 25644136

[12] Weeks A, Zapata F, Pell SK, Daly DC, Mitchell JD, Fine PVA. To move or to evolve: contrasting patterns of intercontinental connectivity and climatic niche evolution in “Terebinthaceae” (Anacardiaceae and Burseraceae). Frontiers in Genetics. 2014;5(409). 10.3389/fgene.2014.00409 25506354 PMC4247111

[13] Zizka A, Carvalho-Sobrinho JG, Pennington RT, Queiroz LP, Alcantara S, Baum DA, et al. Transitions between biomes are common and directional in Bombacoideae (Malvaceae). J Biogeogr. n/a(n/a). 10.1111/jbi.13815

[14] Cantley JT, Markey AS, Swenson NG, Keeley SC. Biogeography and evolutionary diversification in one of the most widely distributed and species rich genera of the Pacific. AoB PLANTS. 2016;8. 10.1093/aobpla/plw043 27339053 PMC4972462

[15] Meudt HM, Rojas-Andrés BM, Prebble JM, Low E, Garnock-Jones PJ, Albach DC. Is genome downsizing associated with diversification in polyploid lineages of *Veronica*? Botanical Journal of the Linnean Society. 2015;178(2):243–66.

[16] Dale EE, Larcombe MJ, Lee WG, Higgins SI. Diversification is decoupled from biome fidelity: *Acacia* a case study. Journal of Biogeography. 2020;47(2):538–52.

[17] Dale EE. The role of biome shifts in lineage diversification. Dunedin, New Zealand: University of Otago; 2019.

[18] Landis MJ, Edwards EJ, Donoghue MJ. Modeling phylogenetic biome shifts on a planet with a past. Systematic biology. 2020. 10.1093/sysbio/syaa045 . 32514540

[19] Eiserhardt WL, Couvreur TLP, Baker WJ. Plant phylogeny as a window on the evolution of hyperdiversity in the tropical rainforest biome. New Phytologist. 2017;214(4):1408–22. 10.1111/nph.14516 28277624

[20] Wardle P. Vegetation of New Zealand. Cambridge, UK: Cambridge University Press; 1991.

[21] Lee DE, Lee WG, Mortimer N. Where and why have all the flowers gone? Depletion and turnover in the New Zealand Cenozoic angiosperm flora in relation to palaeogeography and climate. Australian Journal of Botany. 2001;49(3):341–56.

[22] Wilmshurst JM, Anderson AJ, Higham TF, Worthy TH. Dating the late prehistoric dispersal of Polynesians to New Zealand using the commensal Pacific rat. Proceedings of the National Academy of Sciences. 2008;105(22):7676–80. 10.1073/pnas.0801507105 18523023 PMC2409139

[23] McGlone MS, Newnham RM, Moar NT. The vegetation cover of New Zealand during the Last Glacial Maximum: do pollen records under-represent woody vegetation. Terra Australis. 2010;32:49–68.

[24] Heenan PB, McGlone MS. Evolution of New Zealand alpine and open-habitat plant species during the late Cenozoic. New Zealand Journal of Ecology. 2013;37(1):105–13.

[25] Moncrieff GR, Bond WJ, Higgins SI. Revising the biome concept for understanding and predicting global change impacts. Journal of Biogeography. 2016;43(5):863–73. 10.1111/jbi.12701

[26] Lee DE, Lee WG, Jordan GJ, Barreda VD. The Cenozoic history of New Zealand temperate rainforests: comparisons with southern Australia and South America. New Zealand Journal of Botany. 2016;54(2):100–27. 10.1080/0028825X.2016.1144623

[27] García-Verdugo C, Caujapé-Castells J, Sanmartín I. Colonization time on island settings: lessons from the Hawaiian and Canary Island floras. Botanical Journal of the Linnean Society. 2019;191(2):155–63. 10.1093/botlinnean/boz044

[28] Brandt AJ, Lee WG, Tanentzap AJ, Hayman E, Fukami T, Anderson BJ. Evolutionary priority effects persist in anthropogenically created habitats, but not through nonnative plant invasion. New Phytologist. 2017;215(2):865–76. 10.1111/nph.14544 28407248

[29] Magallón S, Sanderson MJ. Absolute diversification rates in angiosperm clades. Evolution. 2001;55(9):1762–80. 10.1111/j.0014-3820.2001.tb00826.x 11681732

[30] Poole AL, Adams NM. Trees and shrubs of New Zealand. Lincoln, NZ: Manaaki Whenua Press; 1964.

[31] Molloy BPJ, Druce AP. A new species name in *Melicytus* (Violaceae) from New Zealand. New Zealand Journal of Botany. 1994;32(2):113–8. 10.1080/0028825X.1994.10410362

[32] Molloy BPJ, Clarkson BD. A new, rare species of *Melicytus* (Violaceae) from New Zealand. New Zealand Journal of Botany. 1996;34(4):431–40. 10.1080/0028825X.1996.10410124

[33] Heenan PB, de Lange PJ. A new and remarkably local species of *Myrsine* (Myrsinaceae) from New Zealand. New Zealand Journal of Botany. 1998;36(3):381–7. 10.1080/0028825X.1998.9512576

[34] Heenan P, de Lange P. Myrsine aquilonia and *M*. *umbricola* (Myrsinaceae), two new species from New Zealand. New Zealand Journal of Botany. 2004;42(5):753–69.

[35] Mark AF. Above the treeline: a nature guide to the New Zealand mountains: Craig Potton Publishing; 2013.

[36] Edgar E, Connor H. Flora of New Zealand. Vol. V. Lincoln. Manaaki Whenua Press; 2000.

[37] Johnson PN, Brooke P. Wetland plants in New Zealand. Wellington, New Zealand: DSIR Publishing; 1989.

[38] Mark AF, Adams NM. New Zealand alpine plants. Auckland, New Zealand: Godwit Publishing Ltd.; 1995.

[39] Wilson HD. Stewart Island Plants: Manuka Press; 1994.

[40] Wilson HD. Wild plants of Mount Cook National Park: Manuka Press; 1996.

[41] Bayly MJ, Kellow A. An illustrated guide to New Zealand hebes. Wellington, New Zealand: Te Papa Press; 2006.

[42] Wilson HD, Galloway T. Small-leaved shrubs of New Zealand: Manuka Pr.; 1993.

[43] Matzke NJ. BioGeoBEARS: BioGeography with Bayesian (and likelihood) evolutionary analysis in R Scripts. R package, version 02. 2013;1:2013.

[44] Dale EE, Larcombe MJ, Lee WG. Biome occupancy data used in "The effect of single biome occupancy on the estimation of biome shifts and the detection of biome conservatism". Fig Share2020. 10.6084/m9.figshare.13373072 PMC8009365 33784318

[45] Ringelberg JJ, Zimmermann NE, Weeks A, Lavin M, Hughes CE. Biomes as evolutionary arenas: Convergence and conservatism in the trans-continental succulent biome. Global Ecology and Biogeography. 2020;29(7):1100–13. 10.1111/geb.13089.

[46] Orme D, Freckleton R, Thomas G, Petzoldt T, Fritz S, Isaac N, et al. caper: Comparative Analyses of Phylogenetics and Evolution in R. R package version 1.0.1 ed2018.

[47] Fritz SA, Purvis A. Selectivity in mammalian extinction risk and threat types: a new measure of phylogenetic signal strength in binary traits. Conservation Biology. 2010;24(4):1042–51. 10.1111/j.1523-1739.2010.01455.x 20184650

