# Supplementary material for: The effect of single biome occupancy on the estimation of biome shifts and the detection of biome conservatism
Source: PLoS One. 2021 Mar 30;16(3):e0248839. doi: 10.1371/journal.pone.0248839 (PMC8009365; doi:10.1371/journal.pone.0248839)
Supplement: S1 Table — A one-tailed t-test testing the significance of the difference in biome shifts between single and multiple biome approaches for all clades, for all clades and excluding the New Zealand clades, to test whether these clades were overly-influencing results. Significant p-values are indicated with an *. (DOCX) [file pone.0248839.s002.docx]

**S1 Table:** A one-tailed t-test testing the significance of the difference in biome shifts between single and multiple biome approaches for all clades, for all clades and excluding the New Zealand clades, to test whether these clades were overly-influencing results. Significant p-values are indicated with an *.

| **Output** | **All clades** | **NZ clades excluded** | |
| --- | --- | --- | --- |
| df | 17 | 7 |  |
| t | 6.23 | 3.20 |  |
| p-value | <0.001* | 0.007* |  |
