# Supplementary material for: The effect of single biome occupancy on the estimation of biome shifts and the detection of biome conservatism
Source: PLoS One. 2021 Mar 30;16(3):e0248839. doi: 10.1371/journal.pone.0248839 (PMC8009365; doi:10.1371/journal.pone.0248839)
Supplement: S2 Table — Phylogenetic null model analysis testing the effect of single versus multiple biome occupancy on biome conservatism with raw biome shift estimates used for both. Null simulations were conducted for each clade by randomising biomes occupied, with 1000 replicates per clade. Biome conservatism is considered significant (*) if 0.95 or greater of the simulations have more biome shifts than the observed biome shift count. A positive/negative change in tendency towards biome conservatism comparing single with multiple biome occupancy approaches is indicated. (DOCX) [file pone.0248839.s003.docx]

**S2 Table:** Phylogenetic null model analysis testing the effect of single versus multiple biome occupancy on biome conservatism with raw biome shift estimates used for both. Null simulations were conducted for each clade by randomising biomes occupied, with 1000 replicates per clade. Biome conservatism is considered significant (*) if 0.95 or greater of the simulations have more biome shifts than the observed biome shift count. A positive/negative change in tendency towards biome conservatism comparing single with multiple biome occupancy approaches is indicated.

|  | **Proportion of null simulations greater than observed biome shifts** | | **Absolute (percentage) change in tendency towards biome conservatism** |
| --- | --- | --- | --- |
| **Clade** | **Single biome occupancy approach** | **Multiple biome occupancy approach** |  |
| *Chionochloa* | 0.44 | 0.50 | +0.06 (14%) |
| *Coprosma* | 0.41 | 0.42 | +0.01 (2%) |
| *Melicytus* | 0.98* | 0.99* | +0.01 (1%) |
| *Myrsine* | 0.11 | 0.00 | -0.11 (100%) |
| *Poa* X | 0.85 | 0.98* | +0.13 (15%) |
| *Pseudopanax* | 0.77 | 0.76 | -0.01 (1%) |
| *Rytidosperma* A | 0.29 | 0.19 | -0.10 (34%) |
| *Rytidosperma* B | 0.71 | 0.72 | +0.01 (1%) |
| *Veronica* | 0.49 | 0.70 | +0.21 (43%) |
