# Supplementary material for: The effect of single biome occupancy on the estimation of biome shifts and the detection of biome conservatism
Source: PLoS One. 2021 Mar 30;16(3):e0248839. doi: 10.1371/journal.pone.0248839 (PMC8009365; doi:10.1371/journal.pone.0248839)
Supplement: S1 Flow diagram — (DOC) [file pone.0248839.s004.doc]

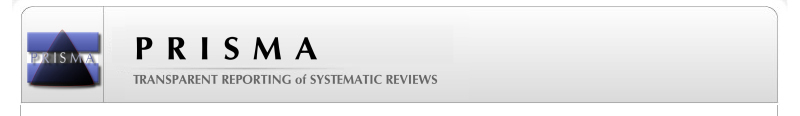
**PRISMA 2009 Flow Diagram**

**Screening**

**Included**

**Eligibility**

**Identification**

Records identified through database searching
(n =0 )

Additional records identified through other sources
(n =43 )

Records after duplicates removed
(n =41 )

Records screened
(n = 41 )

Records excluded
(n = 11 )

Full-text articles assessed for eligibility
(n = 30 )

Full-text articles excluded, with reasons
(n = 17 )

Studies included in qualitative synthesis
(n = 13 )

Studies included in quantitative synthesis (meta-analysis)
(n =13 )
